# Frontal Recess Cells in International Frontal Sinus Anatomy Classification (IFAC); Prevalence, Infection Incidence, and Relation to Frontal Sinus Infection in Chronic Sinusitis Patients

**DOI:** 10.1007/s12070-021-03069-8

**Published:** 2022-01-16

**Authors:** Ahmed Abdelfattah Bayomy Nofal, Mohammad Waheed El-Anwar

**Affiliations:** Otorhinolaryngology-Head and Neck Surgery Department, Faculty of Medicine, Zagazig University, Zagazig, Egypt

**Keywords:** Endoscopic sinus surgery, Frontal recess, Frontal recess cells, Frontal sinus, IFAC

## Abstract

Frontal recess cells have many types with different sizes, arrangement, and extend. It plays an important role in successful functional endoscopic sinus surgery (FESS) as most causes of failure are related to it. Outline the prevalence of the frontal recess cells, pathological incidence of each cell regarding to frontal sinus pathology. Prospective study on 100 consecutive patients (200 sides) complaining from nasal and sinus symptoms which did not respond to medical management and indicated for FESS. Anterior group was infected in 30.8%; agger nasi cell (ANC) present in 97% (25.8% infected, 74.2% not infected), supra agger cell (SAC) present in 48% (39.6% infected, 60.4% not infected), supra agger frontal cell (SAFC) present in 11% (36.4% infected, 63.6% not infected). Posterior group was infected in 24.8%; supra bulla cell (SBC) present in 72% (30.6% infected, 69.4% not infected), supra bulla frontal cell (SBFC) present in 23% (17.4% infected, 82.6% not infected), supra orbital ethmoid cell (SOEC) present in 42% of cases (19% infected, 81% not infected). Medial group [frontal septal cell (FSC)] was present in 21% (33.3% infected, 66.7% not infected). FSC, SAC, SAFC, and SBC showed high infection rate in association with infected frontal sinus, while, the SOEC, ANC, and SBFC did not have such high infection rate. Frontal recess cells show no difference in their prevalence either if the frontal sinus infected or not, however their infection rate show significant difference.

## Introduction

Understanding the anatomy of frontal sinus, frontal sinus drainage, and frontal recess cells is indispensable in planning successful frontal sinus surgery not only to avoid the complications but also to prevent disease recurrence.

The frontal recess cells affect the frontal sinus drainage and have many types with different sizes, arrangement, and extend. They had many different names and descriptions. Many efforts had been done to identify and classify these cells showing its influence on the frontal sinus drainage.

Schaeffer in 1916 was the first one who started to describe these cells and he developed the term frontal cells in order to describe the accessory sinus cells for the frontal sinus [1].

In 1941 Van Alyea defined the frontal cells as the cells encroaching on the frontal recess or the frontal sinus. He divided them to two anatomic types; frontal recess cells, and invading frontal cells [2].

Bent and Kuhn [3] in 1994 define the frontal cells as cells derived from the anterior ethmoid sinus behind and above the agger nasi cell and pneumatize the frontal recess. They excluded agger nasi, supra orbital ethmoid, and intersinus septal cell from the frontal cells. They divided the frontal cells to four different types based on coronal cuts of the CT scan [3].

All the above classifications depended on the cadaveric study and CT scan coronal plain study.

The European Position Paper (EPOS) on the Anatomical Terminology of the Internal Nose and Paranasal Sinuses defined the frontal recess as the most anterosuperior part of the ethmoid, inferior to the frontal sinus opening, and clarified that pneumatization on the frontal recess can extend from the agger nasi, ethmoidal bulla or the terminal recess of the ethmoidal infundibulum [4].

EPOS termed these cells ‘anterior ethmoidal’ cells, if they do not extend into the frontal sinus, but if they do they termed ‘frontoethmoidal’ cells. The EPOS classified the frontoethmoidal cells to anterior, posterior, medial, or lateral, with respect to the frontal recess / inner walls of the frontal sinus [4].

In 2016 the International Frontal Sinus Anatomy Classification (IFAC) was published depending on the triplanar CT scan and it classified the frontal recess cells based on their anatomic origin into three groups [5]. The anterior cells which push the frontal sinus drainage pathway medially, posteriorly or posteromedially, and it include agger nasi cell (ANC), supra agger cell (SAC), and supra agger frontal cell (SAFC). The posterior cells which push the frontal sinus drainage pathway anteriorly, and it include supra bulla cell (SBC), supra bulla frontal cell (SBFC), and supra orbital ethmoid cell (SOEC). The medial cells which push the frontal sinus drainage pathway laterally and it include the frontal septal cell (FSC).

The aim of this study is to outline the prevalence of frontal recess cells based on IFAC and the pathological incidence of each cell with its relation to frontal sinus in chronic sinusitis patients. This is done in to two steps; first by studying the CT scan of patients complaining from nasal and sinus symptoms not responding to medical management, and indicated for surgery by two otolaryngologist. Second step is by reviewing the intraoperative finding during functional endoscopic sinus surgery (FESS) with multiple times revision of the CT scans in triplanner manner intra-operatively.

## Methods

This is a prospective study of 100 consecutive patients (200 sides) who were complaining from at least two of the following symptoms (nasal block, nasal/postnasal discharge, facial pain/pressure, or reduction/loss of smell) for at least 12 weeks in the period from March 2018 to June 2020. They did not improved by the medical treatment and all were operated due to nasal and/or sinus pathology according to the individual pathology of each. Informed written consent was signed by all subjects to share in the study after explanation of its purpose and after approval of the institutional review board.

The assessment of the frontal recess cells and frontal sinus were done in to two steps. First step is by studying none contrast CT scans of the maxillofacial area of the patients in triplanner manner by two separate Otolaryngologists. Each side was judged separately (200 sides). The second step is by evaluating each cell throughout its presence, extending, type, involvement by polyps or pus during FESS with multiple times intraoperative revision of the CT scan in triplanner manner.

Frontal recess cell or frontal sinus is considered infected if there is opacification or mucosal thickening within the sinus or the cells in the CT scan confirmed by presence of pus or polypoidal mucosa during FESS.

All the CT scans were done just after medical management to eliminate acute infection and control the allergic symptoms before doing the CT scan. The patient less than 16 years old, previous nasal surgery, suspected nasal or sinus neoplasm were excluded from the study.

The CT scans were done with a 64 slice CT scan (Light speed volume VCT, GE medical system, Milwaukee, WI, USA). The protocol of 64-slice MDCT was performed with a 0.625 mm detector width, a 1 mm section width and a 0.5 mm interval reconstruction.

Axial, coronal, and sagittal reformatted CT scan were reviewed in synapse Fujifilm's medical imaging and information management system, SYNAPSE with comprehensive PACS (Picture Archiving and Communication System) which allowing simultaneous viewing of the CT scan in axial, coronal, and sagittal planes with localizing ability.

All the observations were performed independently by two readers (the authors). If there is any difference they reviewed the scans together to reach a consensus and confirmed with the second step by evaluating of the cell during FESS with multiple times intraoperative revision of the CT scan in triplanner manner. The data was collected, tabulated and analyzed.

**Fig. 1:**
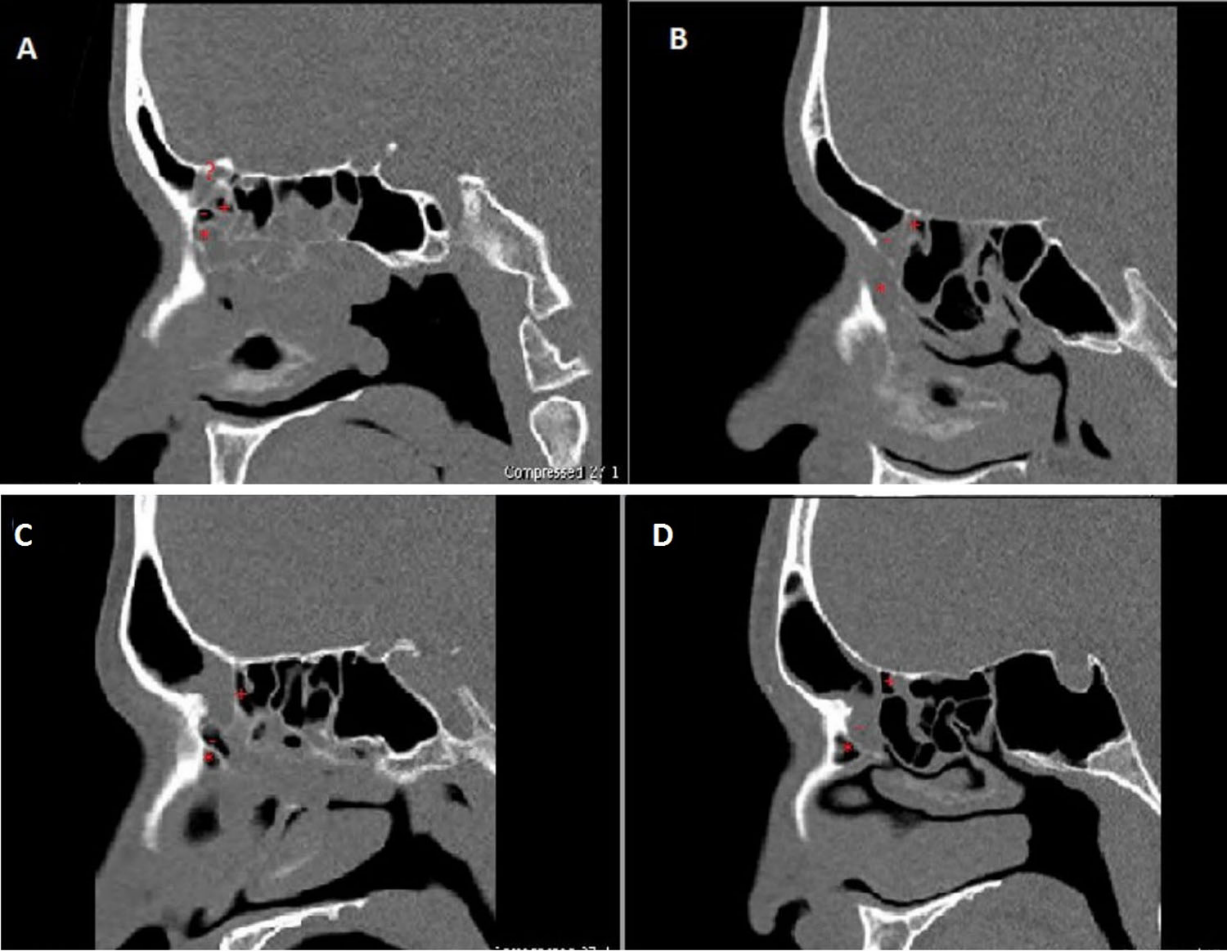
**A** Not infected frontal sinus with infected agger nasi cell (*) and supra bulla frontal cell (?), not infected supra agger cell (−) and supra bulla cell (+). **B** Not infected frontal sinus with infected agger nasi cell (*) and supra agger cell (−) with not infected supra bulla cell (+). **C:** Frontal sinusitis with not infected agger nasi cell (*), supra agger cell (−), supra bulla cell (+). **D** Frontal sinusitis with not infected aggernasi cell (*), supra bulla cell (+), and infected supra agger cell (−)

The frontal recess cells were studied based on IFAC [5]. The anterior cells include agger nasi cell (ANC), supra agger cell (SAC), and supra agger frontal cell (SAFC). The posterior cells include supra bulla cell (SBC), supra bulla frontal cell (SBFC), and supra orbital ethmoid cell (SOEC). The medial cells include the frontal septal cell (FSC) (Fig. 1).

Statistical analysis was accompanied with the SPSS statistical software package (version 25; SPSS, Inc., Chicago, IL, USA). *P* value of < 0.05 was considered statistically significant.

## Results

This study included 100 patients (200 sides), 67 male (67%), and 33 female (33%). The patients’ age ranged from 16 to 62 years (42.6 ± 13.2).

The anterior group cells represented the most common prevalence group of cells; there total number was 312 cells from which 96 cells were infected (30.8%) and 216 cells were not infected (69.2%). The ANC was the most common cell as it was present in 97% of cases from which 25.8% were infected and 74.2% were not infected. The SAC was present in 48% of cases from which 39.6% were infected and 60.4% were not infected. The SAFC was the least presented cell, only in 11% of the cases from which 36.4% were infected and 63.6% were not infected (Table 1).

**Table 1:** The prevalence and infection rate of the frontal recess cells

	Total number of cells	Infected cells (N1)	Non infected cells (N2)	*P* value
*Anterior cells*	312	96 (30.8%)	216 (69.2%)	0.0474 S (X^2^ = 6.098)
ANC	194 (97%)	50 (25.8%)	144 (74.2%)	
SAC	96 (48%)	38 (39.6%)	58 (60.4%)	
SAFC	22 (11%)	8 (36.4%)	14 (63.6%)	
*Posterior cells*	274	68 (24.8%)	206 (75.2%)	0.06723912 NS (X^2^ = 5.399)
SBC	144 (72%)	44 (30.6%)	100 (69.4%)	
SBFC	46 (23%)	8 (17.4%)	38 (82.6%)	
SOEC	84 (42%)	16 (19%)	68 (81%)	
*Medial cells*				
FSC	42 (21%)	14 (33.3%)	28 (66.7%)	

*ANC* aggernasi cell, *SAC* supra agger cell, *SAFC*, supraagger frontal cell, *SBC* supra bulla cell, *SBFC* supra bulla frontal cell, *SOEC* supra orbital ethmoid cell, *FSC* frontal septal cell, *S* significant, *NS* non-significant

The posterior group cells total number was 274 cells from which 68 cells were infected (24.8%) and 206 cells were not infected (75.2%). The SBC was the commonest cell and present in 72% of cases from which 30.6% were infected and 69.4% were not infected. The SBFC was the least presented, found in 23% of cases from which 17.4% were infected and 82.6% were not infected. The SOEC present in 42% of cases from which 19% were infected and 81% were not infected (Table 1).

The medial group cell (FSC) present in 21% of cases from which 33.3% were infected and 66.7% were not infected (Table 1).

**Fig. 2:**
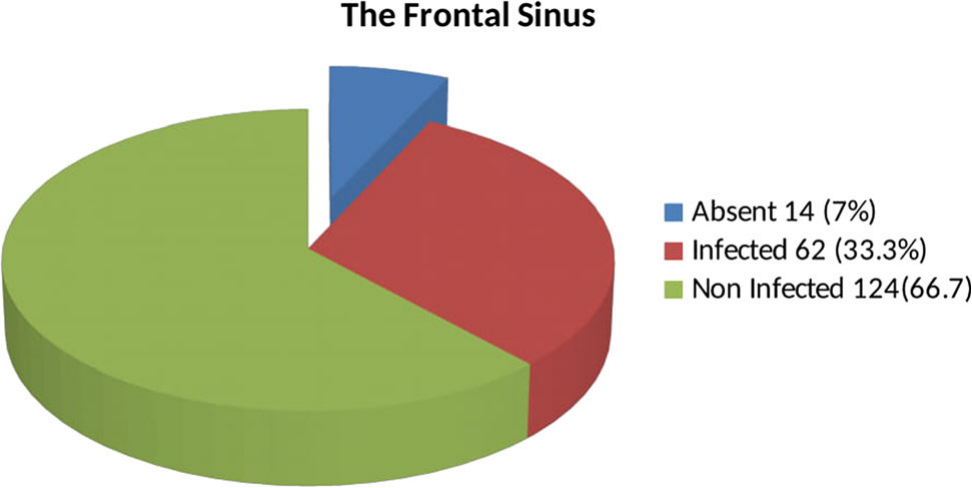
The prevalence and infection rate of the frontal sinus

The frontal sinus was absent in 14 case (7%) and present in 186 cases (93%) from which it was infected in 62 case (33.3%) and not infected in 124 case (66.7%) (Fig. 2).

In the cases where the frontal sinus were infected (62 cases), the frontal recess was infected in 90.3% of the case while in 9.7% cases it was not infected. The most prevalence cell was the ANC which was found in 96.8% (50% infected and 50% not infected). The SAC was found in 54.9% of cases (70.6% infected and 29.4% not infected). The SAFC was present in 19.4% of cases (66.7% infected and 33.3% not infected). The SBC was present in 80.6% of cases (64% infected and 36% not infected). The SBFC was present in 22.6% of cases (28.6% infected and 71.4% not infected). The SOEC was present in 41.9% of cases (53.8% infected and 46.1% not infected). The FSC was present in 22.6% of cases all were infected (100%) (Table 2).

**Table 2:** The prevalence and infection rate of frontal recess cells in infected frontal sinus cases

Total number of cells	Frontal Recess		Anterior cells						Posterior cells							Medial cells
			ANC		SAC		SAFC		SBC		SBFC		SOEC		FSC	
			60		34		12		50		14		26		14	
Infection	+	−	+	−	+	−	+	−	+	−	+	−	+	−	+	−
	56	6	30	30	24	10	8	4	32	18	4	10	14	12	14	0
Percent of cell prevalence	100%		96.8%		54.9%		19.4%		80.6%		22.6%		41.9%		22.6%	
Percentof infection	90.3%	9.7%	50%	50%	70.6%	29.4%	66.7%	33.3%	64%	36%	28.6%	71.4%	53.8%	46.1%	100%	0%

+ , infected cells; −, not infected cells; ANC, aggernasi cell; SAC, supra agger cell; SAFC, supra agger frontal cell; SBC, supra bulla cell; SBFC, supra bulla frontal cell; SOEC, supraorbital ethmoid cell; FSC, frontal septal cell

In the cases where the frontal sinus were not infected (124 cases) or absent (14 cases) (total 138 cases), the frontal recess was infected in 23.2% and not infected in 76.8% of cases. The ANC was the most prevalence cell and found in 97.1% (14.9% infected and 85.1% not infected). The SAC was found in 44.9% of cases (22.6% infected and 77.4% not infected). The SAFC was present in 7.2% of cases all not infected (100%). The SBC was present in 68.1% of cases (12.8% infected and 87.2% not infected). The SBFC was present in 23.2% of cases (12.5% infected and 87.5% not infected). The SOEC was present in 42% of cases (3.5% infected and 96.5% not infected). The FSC was present in 22.6% of cases all were not infected (100%) (Table 3).

**Table 3:** The prevalence and infection rate of frontal recess cells in not infected frontal sinus cases

Total number of cells	Frontal recess		Anterior cells						Posterior cells						Medial cells	
			ANC		SAC		SAFC		SBC		SBFC		SOEC		FSC	
			134		62		10		94		32		58		28	
Infection	+	−	+	−	+	−	+	−	+	−	+	−	+	−	+	−
Number	32	106	20	114	14	48	0	10	12	82	4	28	2	56	0	28
Percent of cell prevalence	100%		97.1%		44.9%		7.2%		68.1%		23.2%		42%		20.2%	
Percent to infected FS	23.2%	76.8%	14.9%	85.1%	22.6%	77.4%	0%	100%	12.8%	87.2%	12.5%	87.5%	3.5%	96.5%	0%	100%

+ , infected cells; −, not infected cells; ANC, aggernasi cell; SAC, supra agger cell; SAFC, supra agger frontal cell; SBC, supra bulla cell; SBFC, supra bulla frontal cell; SOEC, supraorbital ethmoid cell; FSC, frontal septal cell

**Fig. 3:**
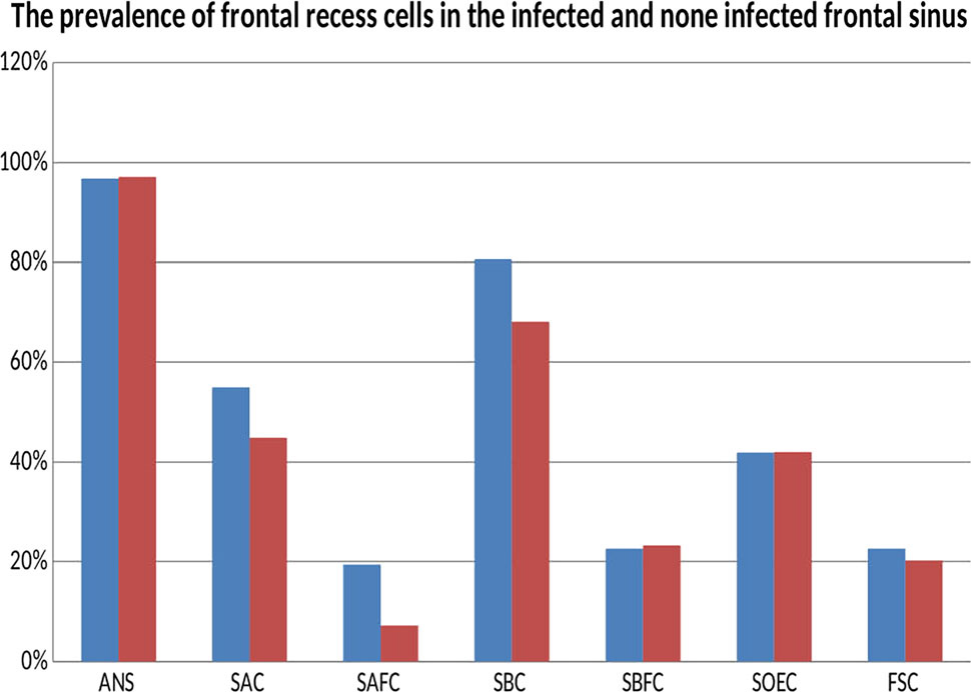
The prevalence of frontal recess cells in infected (bue) and not infected (red) frontal sinus

The prevalence of frontal recess cells in infected and not infected frontal sinus did not show significant difference (Fig. 3).The ANC was present in 97% of the infected frontal sinus cases and nearly the same number (97.1%) in non-infected frontal sinus cases. The SAC was present in 54.9% of infected frontal sinus cases and 44.9% in non-infected frontal sinus cases. The SAFC was present in 19.4% of infected frontal sinus cases and 7% in not infected frontal sinus cases. The SBC was present in 81% of infected frontal sinus cases and 68.1% in not infected frontal sinus cases. The SBFC was present in 22.6% of infected frontal sinus cases and 23.2% in not infected frontal sinus cases. The SOEC was present in 42% in both infected and not infected frontal sinus cases. The FSC was present in 23% of infected frontal sinus cases and 20% in not infected frontal sinus cases.

**Fig. 4:**
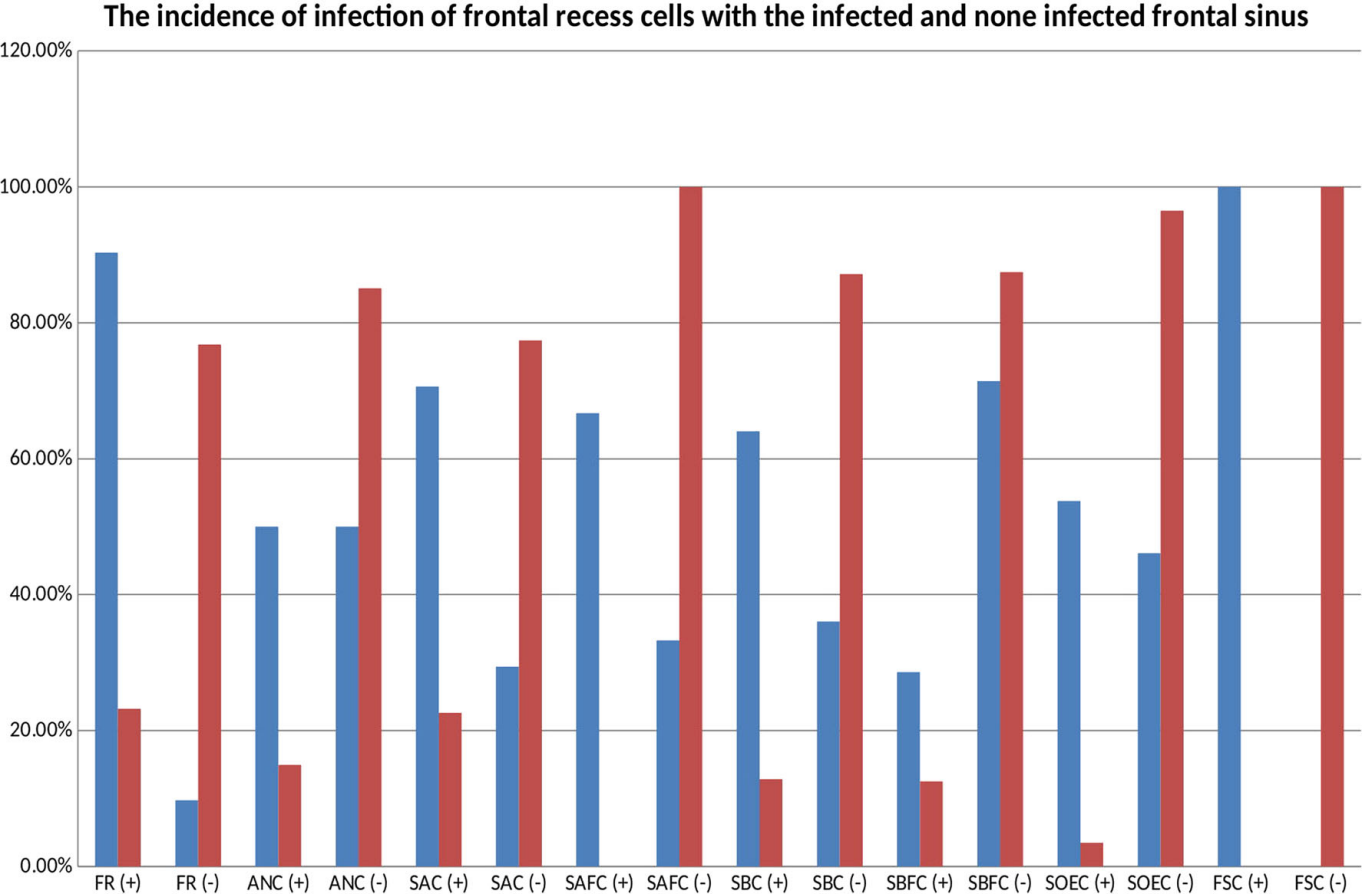
The infection rate of frontal recess cells in infected and not infected frontal sinus

The infection rate of the frontal recess cells in the infected frontal sinus showed significant difference (Fig. 4). The ANC did not show any difference and it was infected in 50% of the cases, and not infected in the other 50%. The SAC was infected 70.6% of the cases and not infected in 29.4% of the cases. The SAFC was infected in 66.7% of the cases and not infected in 33.3% of the cases. The SBC was infected in 64% of the cases and not infected in 36% of the cases. The SBFC was infected in 28.6% of the cases and not infected in 71.4% of the cases. The SOEC was infected in 53.8% of the cases and not infected in 46.1% of the cases. The FSC was infected in 100% of the cases.

So the frontal recess cells FSC (100%), SAC (70.6%), SAFC (66.7%), and SBC (64%) showed high infection rate in association with infected frontal sinus. While, the SOEC (53.8%), ANC (50%), and SBFC (28.6%) did not have such high infection rate with infected frontal sinus (Fig. 4).

The infection rate of the frontal recess cells in non-infected frontal sinus also showed significant difference (Fig. 3). The ANC was infected in 14.9% of cases and not infected in 85.1% of cases. The SAC was infected 22.6% of the cases and not infected in 77.4% of the cases. The SAFC was not infected in 100% of the cases. The SBC was infected in 12.8% of the cases and not infected in 87.2% of the cases. The SBFC was infected in 12.5% of the cases and not infected in 87.5% of the cases. The SOEC was infected in 3.5% of the cases and not infected in 96.5% of the cases. The FSC was not infected in 100% of the cases.

So the frontal recess cells; FSC (100%), SAFC (100%), SOEC (96.5%), SBFC (87.5), SBC (87.2%), and ANC (85.1%) had very high none infection rate in non-infected frontal sinus. However, the SAC (77.4%) showed high none infection rate in non-infected frontal sinus but not as much as others (Fig. 4).

The differences between the rate of infections among different frontal cells types in the anterior group showed significantly higher rate of infection rate among the ANC (X^2^ = 6.098, *P* = 0.0474) while the differences in the infection rates among different frontal cells types in the posterior group were non-significant (X^2^ = 5.399, *P* = 0.067).

## Discussion

The main aim of endoscopic sinus surgery (ESS) is to relieve the patient symptoms that did not respond to maximal medical therapy [5].

The frontal recess cells play an important role in successful endoscopic frontal sinus surgery (EFSS).

The main causes of failure of EFSS are incomplete removal of the frontal recess cells, miss identification of the frontal sinus ostium, recurrence of the mucosal disease, iatrogenic injury to the frontal recess or the frontal sinus drainage area [6, 7].

Most of the above mentioned causes of failure of EFSS are related either directly or indirectly to the frontal recess cells and their orientation and configuration within the frontal recess beside their relation to the frontal sinus ostium and neighbor structures. So, carful understanding of frontal recess cells and their relations is crucial in doing successful EFSS.

The IFAC is new, simple and well defined classification of the frontal recess cells that was published in 2016, and attains growing popularity by time [5]. IFAC classified the frontal recess cells based on their anatomic origin into three groups (anterior, posterior, and medial) [5].

To date, there are only three studies [8–10] documenting the prevalence of frontal recess cells according to IFAC, none of them provide any data of the infection rate of those frontal recess cells (Table 4).

**Table 4:** The prevalence of frontal recess cells according to IFAC in various studies

Authors	Study patient & method	Anterior group cells			Posterior group cells			Medial Group CELLS (FSC) (%)
		ANC (%)	SAC (%)	SAFC (%)	SBC (%)	SBFC (%)	SOEC (%)	
Choby et al. [8]	100 non diseased CT scan – 200 sides	96.5	30	20	72	5.5	28.5	30
Sommer et al [9]	249 patients with sinus symptoms—the patient to be "1" in the study	95.2	49	24.9	88.8	26.5	9.2	27.7
Tran et al. [10]	114 CT scans without frontal sinusitis—208 sides	95.7	16.3	13	46.2	4.3	17.3	10.6
Nofal& El-Anwar (current study)	100 patients with sinus symptoms—200 sides	97	48	11	72	23	42	21

*CT* computed tomography, *ANC* aggernasi cell, *SAC* supra agger cell, *SAFC* supraagger frontal cell, *SBC* supra bulla cell, *SBFC* supra bulla frontal cell, *SOEC* supra orbital ethmoid cell, *FSC* frontal septal cell

Choby et al. [8] at 2018 studied 100 non diseased maxillofacial CT scan, and recorded the following; ANC present in 96.5% of cases, SAC in 30%, SAFC in 20%, SBC in 72%, SBFC in 5.5%, SOEC in 28.5%, and FSC in 30%.

Sommer et al. [9] at 2019 studied the CT scan of 249 patients with sinus symptoms and showed the prevalence rate as follow; ANC present in 95.2% of cases, SAC in 49%, SAFC in 24.9%, SBC in 88.8%, SBFC in 26.5%, SOEC in 9.2%, and FSC in 27.7%. They consider the patient to be "1" in the study if the cell either detected unilaterally or bilaterally [9].

Tran et al. [10] at 2019 studied the CT scan of 114 patients with no frontal sinus disease to assess the prevalence of frontal recess cells and the most common frontal sinus drainage pathways. It shows that; ANC present in 95.7% of cases, SAC in 16.3%, SAFC in 13%, SBC in 46.2%, SBFC in 4.3%, SOEC in 17.3%, and FSC in 10.6%.

In this study, we studied 100 consecutive patients (200 sides) with chronic sinusitis symptoms not respond to medical management. The prevalence and infection rate of frontal recess cells were investigated; when the frontal sinus infected, and when it is not involved in the infection using the triplanner study of CT scans and supported by the intraoperative finding during FESS with multiple times revision of the CT scan in triplanner manner during FESS. It shows; ANC present in 97% of cases, SAC in 48%, SAFC in 11%, SBC in 72%, SBFC in 23%, SOEC in 42%, and FSC in 21%.

In the studies of Choby et al. [8] and Tran et al. [10], they studied none diseased CT scans. Even they excluded any CT scan with sinonasal mucosal thickening. Although it is easier to detect the type and extend of each cell in the non-diseased frontal recess cells, but it cannot reflect the true prevalence especially in the diseased group. They also used the third reviewer judgment for the doubtful cases.

However in the current study, we used intraoperative finding during FESS and multiple times revision of the CT scan in triplanner manner during the operation to judge any doubtful case, which we think it is more accurate and precise.

In the study of Sommer et al. [9], they consider the patient to be "1" in the study and if the cell either detected unilaterally or bilaterally, while in our study we record each side separately which is more accurate to detect the prevalence. Choby et al. [8] and Tran et al. [10] also record each side separately.

In our study, we did not only report the prevalence but also the infection rate of each cell type in relation to the total cases, when the frontal sinus is diseased, and when it is not.

The anterior group has the highest infection rate 31% of the total cases; ANC is 26%, SAC is 40%, and SAFC is 36%. The posterior group has infection rate 25% of total cases; SBC is 31%, SBFC is 17%, and SOEC is 19%. The medial group (FSC) has 33% infection rate.

In infected frontal sinus, the frontal recess cells were found to be frequently affected; FSC is infected 100%, SAC (70.6%), SAFC (66.7%), and SBC (64%), SOEC (53.8%), ANC (50%), and SBFC (28.6%). So, revising each of this cell type prior during FESS is important in to avoid residual disease. The SAC showed significant more incidence of infection reflecting the importance of its routine evaluation during FESS.

In not infected frontal sinus, the ANC is infected in 14.9%, SAC is infected in 22.6%, SAFC is infected in 0%, SBC is infected in 12.8%, SBFC is infected in 12.5%, SOEC is infected in 3.5%, and FSC is infected in 0% of the cases. Thus, even in non-infected frontal sinus, particular attention and revising the SAC, ANC, SBC, SBFC is recommended.

So scrutiny triplanner CT scans study of FESS patients preoperatively and intra-operatively is crucial to identify and clear each infected frontal recess cell individually either the frontal sinus is infected or not. This will play important role in successful FESS not only in avoiding the complications but also preventing disease recurrence. However, further studies with large number of patient in multi centers are still required.

## Conclusion

Frontal recess cells show no difference in their prevalence either if the frontal sinus infected or not, however their infection rate show significant difference. There are some frontal recess cells which showed high infection rate in association with infected frontal sinus rather than other cells, and also there are some cells which have very high none infection rate in non-infected frontal sinus rather than other cells. Nevertheless prudent identification and clearing infected frontal recess cells either the frontal sinus is infected or not is indispensable in successful endoscopic sinus surgery not only to avoid the complications but also to prevent disease recurrence. 
